# The Interplay of Sleep Quality, Mental Health, and Sociodemographic and Clinical Factors among Italian College Freshmen

**DOI:** 10.3390/jcm13092626

**Published:** 2024-04-29

**Authors:** Jessica Dagani, Chiara Buizza, Herald Cela, Giulio Sbravati, Giuseppe Rainieri, Alberto Ghilardi

**Affiliations:** 1 Department of Clinical and Experimental Sciences, University of Brescia, Viale Europa 11, 25123 Brescia, Italy; j.dagani@studenti.unibs.it (J.D.); chiara.buizza@unibs.it (C.B.); giulio.sbravati@unibs.it (G.S.); giuseppe.rainieri@unibs.it (G.R.); 2 Department of Psychology, University of Graz, Universitätsplatz 2, 8010 Graz, Austria

**Keywords:** sleep, college freshmen, mental health, well-being, psychological distress

## Abstract

**Background/Objectives**: Sleep and mental health are closely linked, with sleep deprivation increasing the risk of mental health problems in college students. This study aimed to analyze the role of sleep in the mental health status of a sample of Italian freshmen, considering various mental health outcomes and potential interactions between sleep and other relevant factors, such as sociodemographic characteristics, academic experiences, and mental health history. **Methods**: All freshmen from a medium-sized Italian university were invited to participate in a multidimensional online survey (n = 3756). Sleep quality was assessed through questions on average hours of sleep per night and on satisfaction of perceived sleep quality. Mental health outcomes included psychophysical well-being, psychological distress, substance use, and problematic internet use. Statistical analysis involved multivariate analysis of variance, followed by pairwise comparisons. **Results**: The sample (n = 721) exhibited low levels of well-being and a high prevalence of psychological distress (52.1%). Approximately one-third of students (n = 258) were dissatisfied with their sleep quality, and one-fourth (n = 186) reported inadequate sleep (less than 7 h per night). More specifically, 24.4% of students slept on average six hours per night, and 1.4% slept five hours or less. Satisfaction with perceived sleep quality significantly influenced well-being, psychological distress, and cannabis use (η_p_^2^ = 0.02). Interaction effects were observed between satisfaction with sleep quality and drop-out intentions (η_p_^2^ = 0.01), as well as between satisfaction with sleep quality and history of mental health diagnosis (η_p_^2^ = 0.02), both of which were significant for psychological distress and cannabis use. **Conclusions**: This study highlights the influence of perceived sleep quality on academic distress among college freshmen, particularly those with higher intentions of leaving university and with a history of mental health diagnosis.

## 1. Introduction

Mental health issues are prevalent among university students, with the academic environment introducing various stressors, such as increased workload and lifestyle adjustments, including changes in sleep and eating habits [1,2]. Indeed, poor sleep hygiene is prevalent among college students, characterized by insufficient sleep duration and low sleep quality [3,4]. This might be particularly pronounced among freshmen, who are often more vulnerable to the challenges of adapting to the new stressful academic environment [5,6]. A recent European cross-sectional study found support for this, showing a higher prevalence of insomnia symptoms among newer university students [7].

Students’ mental health is influenced by a myriad of factors, including sociodemographic characteristics, mental health history, academic experience, and lifestyle habits. For instance, gender and relationship status have been associated with stress levels among Australian nursing students [8], while students with a family or personal history of mental illness are at higher risk of developing mental health problems in university [9]. Moreover, students who opt to leave university often grapple with mental health challenges [10]. Research also indicates a correlation between mental health symptoms and poor sleep [3,11], with sleep problems in college students having serious consequences, including cognitive deficits, risky behaviors, impaired relationships, and overall poor health [12,13]. Two large-scale studies involving American college students found significant associations between anxiety and depression symptoms with poor sleep quality [14,15]. Additionally, a meta-analysis by Scott and colleagues [16] on sleep-improvement interventions suggested a causal relationship between sleep and mental health difficulties. Moreover, insomnia has been linked to decreased academic performance and unhealthy behaviors [4,17], which are highly prevalent among university students, including problematic internet use and alcohol/cannabis misuse [18,19,20]. While research on problematic internet use and its associations with sleep has primarily focused on adolescents [21,22], some studies on university students have reported similar significant associations [23,24]. Concerning alcohol consumption, evidence suggests that it may exacerbate sleep problems [25], although findings from university student cohorts are not entirely consistent [6,11]. Similarly, hazardous cannabis use has been linked to insomnia symptoms among college students [26,27], but further research is needed to better understand the nature of this association.

The aim of the present study was to explore the role of sleep in the mental health status of a large sample of Italian freshmen, considering various mental health outcomes and potential interactions with sleep and sociodemographic characteristics, health history, and academic factors. 

## 2. Materials and Methods

### 2.1. Study Design

This cross-sectional observational study involved freshmen from a medium-sized university in northern Italy. Data collection took place from May to June 2022. In collaboration with the University administration, an email invitation was sent to all freshmen enrolled in the academic year 2021/2022 to participate in a multidimensional online survey created with LimeSurvey (www.limesurvey.org), a tool ensuring completely anonymous data collection. The email included a description of the study and a link to access the survey. Upon accessing the web link, participants were asked to provide informed consent before proceeding with the survey. LimeSurvey automatically removed any participant identifiers from the survey data, delivering only deidentified data to the investigators. Organizational ethics approval was obtained from the Board of Directors of the University of Brescia (approved with provision no. 330 on 22 November 2021). The survey was conducted in accordance with the World Medical Association’s Helsinki Declaration for Human Studies.

### 2.2. Survey Instrument

This multidimensional survey assessed a wide range of sociodemographic and academic characteristics, as well as multiple aspects related to students’ psychological and physical well-being. Sleep quality was assessed through two questions: a question about the average number of hours slept per night and another on the satisfaction with perceived sleep quality. More specifically, students were asked the following open-ended question: “How many hours of sleep do you get per night on average?” and were asked to estimate their usual sleep duration to the nearest whole hour. The number of hours slept per night was further dichotomized into two categories: “less than seven hours” and “seven or more hours”. Satisfaction with perceived sleep quality was measured on a five-point Likert scale ranging from 0 (“Very dissatisfied”) to 4 (“Very satisfied”), with participants later classified into three categories based on their answers: Dissatisfied (0 = “Very dissatisfied” or 1 = “Quite dissatisfied”), Neither satisfied nor dissatisfied (2 = “Neither satisfied nor dissatisfied”), and Satisfied (3 = “Quite dissatisfied” or 4 = “Very dissatisfied”). In our analyses, we primarily focused on two of these categories: the Dissatisfied students and the Satisfied students. We specifically focused on students who clearly and distinctly expressed either satisfaction or dissatisfaction with their quality of sleep (as opposed to those who appeared neutral) in order to more precisely assess the impact of this satisfaction on mental health outcomes. 

To evaluate students’ mental health status, we included measures of psychophysical well-being and psychological distress related to academic stressors as outcome measures. We opted for psychophysical well-being as it encompasses a broad range of emotional and psychological experiences of college students, and we included psychological distress related to academic stressors as a construct specific to our sample, given the constant pressures of academic demands experienced by college students [28]. Additionally, we incorporated measures related to prevalent unhealthy behaviors among students, such as problematic internet use and the use of alcohol and cannabis. The inclusion of these measures provided a more comprehensive approach to mental health status, considering their high prevalence among students and their impact on several mental health aspects [23,29,30,31,32].

More specifically, the survey included the following standardized tools:

- General Health Questionnaire (GHQ-12) [33]. The GHQ-12 is a widely used self-administered rating scale assessing mental health and psychophysical well-being. It comprises 12 questions with a four-point response scale. In this study, we used the standard bimodal method (0-0-1-1) of scoring, where a score of 0 is assigned to the first two low-stress alternatives and a score of 1 is given to the two high-stress alternatives. Total scores range from 0 to 12, with scores above 3 indicating psychological distress. The GHQ-12 showed good reliability in our sample, as indicated by a Cronbach’s alpha value of 0.78.
- University Stress Scale (USS) [34]. The USS is a 21-item screening test measuring the cognitive appraisal of demands across the range of environmental stressors experienced by students. Each item is rated on a four-point Likert scale (0 = “Not at all”, 3 = “Constantly”). The sum of all items gives the extent score, ranging from 0 to 63. An extent score of 13 or higher indicates significant psychological distress. The USS showed high reliability in our sample, as measured by a Cronbach’s alpha value of 0.85.
- A modified version of World Health Organization-ASSIST v3.0 (ASSIST). The ASSIST is a questionnaire aimed at evaluating substance use for ten different substances: tobacco, alcohol, cannabis, cocaine, amphetamine-type stimulants, inhalants, sedatives, hallucinogens, opioids, and “other drugs”. A score is determined for each substance and categorized as low (occasional or nonharmful use), moderate (more regular use or harmful/hazardous use), or high-risk use (frequent high-risk use or suggestive of dependence). In this study, we employed the self-report adaptation of Barreto and colleagues [35] and focused on alcohol and cannabis use patterns, due to their prevalence among students. Participants were categorized as low-risk or moderate/high-risk users based on established cut-off scores (10 for alcohol and 3 for cannabis). The ASSIST’s ability to classify drug use severity has been extensively validated [36,37]. In our sample, Cronbach’s alpha values were confirmed at 0.56 for alcohol and at 0.79 for cannabis.
- Internet Abusive Use Questionnaire (IAUQ) [38]. The IAUQ assesses problematic internet use and includes 12 items rated on a five-point Likert scale (0 = “totally disagree”, 4 = “totally agree”). The total score ranges from 0 to 48 and the author suggested a cut-off of 24 as indicative of problematic internet use. We categorized participants based on their total score: nonproblematic internet use (below the cut-off) and problematic internet use (above the cut-off). In this sample, the IAUQ demonstrated high reliability, as measured by a Cronbach’s alpha value of 0.90.

### 2.3. Data Analysis

This study aimed to explore the relationship between sleep patterns and mental health outcomes among college students. The analysis included correlation analysis, multivariate analysis of variance (MANOVA), and follow-up univariate analyses (ANOVAs) with pairwise comparisons.

Descriptive statistics were computed for sociodemographic and clinical characteristics, as well as for questionnaire scores. Categorical variables were summarized using percentage distributions, while means and standard deviations (SD) were used for quantitative variables.

Prior to MANOVA, correlations among the dependent variables were examined to assess multicollinearity. Pearson correlation coefficients were calculated between the mental health measures (outcome variables), including USS, GHQ-12, ASSIST for alcohol and cannabis use, and IAUQ.

A MANOVA was performed to assess the overall differences in mental health outcomes across groups defined by the predictors, including sleep patterns (perceived sleep quality and average hours of sleep per night) and demographic factors (gender, employment status, relationship status, field of study, drop-out intentions, lifetime psychological support, and lifetime mental health diagnosis). Pillai’s Trace was chosen among the multivariate tests due to its conservative nature and robustness to violations of assumptions.

Significant results from the MANOVA were followed up with univariate analyses (ANOVAs) to examine the effects of individual predictors on each mental health outcome separately. Bonferroni correction was applied to adjust for multiple comparisons when conducting pairwise comparisons between groups.

Pairwise comparisons were conducted using estimated marginal means to examine differences between specific groups or levels of the predictors. Bonferroni correction was applied to control the family-wise error rate in the pairwise comparisons.

All statistical analyses were conducted using IBM SPSS Statistics (version 29). The significance level was set at α = 0.05 for all tests.

## 3. Results

### 3.1. Characteristics of the Sample

Among the 3756 freshmen in the study population, 721 (19.2%) completed the survey. The participants had a mean age of 20.83 years (SD = 3.83), and the majority (63.4%) were female. Most students (70.6%) had never received psychological support, and only 6.1% had ever received a diagnosis of a mental disorder. Notably, a high proportion of students (81.3%) scored above the cut-off point on the GHQ-12, indicating low levels of psychophysical well-being. Similarly, over half of the participants (52.1%) scored in the range indicating significant psychological distress on the USS. Regarding sleep patterns, 24.4% of students indicated sleeping an average of six hours per night, while only a limited number (1.4%) indicated sleeping five hours or less. Conversely, only three participants reported sleeping more than nine hours per night. Additional sample characteristics are reported in Table 1.

### 3.2. Multivariate Analysis

Statistical analysis showed significant correlations between the dependent variables (Pearson coefficients for significant correlations ranging from 0.089 to 0.423; see Supplementary Materials Table S1), supporting the rationale for conducting a MANOVA to further explore the relationships and differences across groups defined by the predictors. Table 2 displays significant multivariate effects observed for predictors included in the analysis, such as gender, satisfaction with perceived quality of sleep, lifetime psychological support, lifetime diagnosis of mental disorder, and drop-out intentions. In addition, significant effects were detected also for the interactions of sleep satisfaction both with drop-out intentions and with the lifetime diagnosis of a mental disorder. 

Univariates with post hoc analysis revealed significant differences among students reporting different levels of sleep satisfaction in GHQ-12 total score (F(2, 672) = 3.79, *p* = 0.023, η_p_^2^ = 0.01), USS total score (F(2, 672) = 3.92, *p* = 0.020, η_p_^2^ = 0.01), and ASSIST Cannabis total score (F(2, 672) = 8.65, *p* < 0.001, η_p_^2^ = 0.02). Specifically, students dissatisfied with their sleep quality showed a higher GHQ-12 total score compared with students neither satisfied nor dissatisfied (mean difference = 3.92 points, SE = 1.25, *p* = 0.026, 95% CI [0.29, 6.29]). Moreover, students dissatisfied with their sleep quality exhibited significantly higher USS total scores compared with satisfied students (mean difference = 4.20 points, SE = 1.68, *p* = 0.039, 95% CI [0.16, 8.24]). In addition, students dissatisfied with their sleep quality demonstrated significantly higher ASSIST Cannabis total scores compared with both satisfied students (mean difference = 2.196 points, SE = 0.73, *p* = 0.008, 95% CI [0.45, 3.94]) and neither satisfied nor dissatisfied students (mean difference = 2.799 points, SE = 0.709, *p* < 0.001, 95% CI [1.10, 4.50]).

### 3.3. Interactions among Perceived Sleep Quality and Other Factors

In exploring interactions with pairwise comparisons, we report here only significant differences between students dissatisfied versus students satisfied with their sleep quality. For the other comparisons, refer to Supplementary Materials (Table S2). 

The interaction between satisfaction with perceived sleep quality and drop-out intentions showed significant differences in USS total score (F(2, 672) = 8.29, *p* < 0.001, η_p_^2^ = 0.02). Specifically, among students with medium/high intentions to drop out, those dissatisfied with their sleep quality had a mean USS total score that was 7.51 points higher compared with those satisfied with their sleep quality (SE = 1.98, *p* < 0.001, 95% CI [2.76, 12.27]).

The interaction between satisfaction with perceived sleep quality and lifetime mental health diagnosis showed significant differences in ASSIST Cannabis total score (F(2, 672) = 7.56, *p* < 0.001, η_p_^2^ = 0.02). Specifically, among students who have had a diagnosis of a mental disorder, those dissatisfied with their sleep quality had a mean ASSIST Cannabis total score that was 4.48 points higher compared with those satisfied with their sleep quality (SE = 1.35, *p* = 0.003, 95% CI [1.23, 7.73]).

## 4. Discussion

This study aimed to explore the aggregate effect of sleep on various mental health outcomes among college freshmen, both independently and in interaction with other variables. Results from descriptive analysis supported previous findings on the poor mental health status of college students [39,40,41] and confirmed the prevalence of sleep problems previously highlighted in similar samples [3,4]. Considering sleep duration guidelines which recommend at least seven hours per night for young adults [42], one in four students in our sample did not get enough sleep and about one in three was not satisfied with their sleep quality. Our results contrast with those of previous Italian and European studies, where approximately half (or more) of students reported insufficient sleep quality [43,44,45]. This discrepancy may be attributed to methodological differences, as those studies employed standardized sleep assessment tools while our study relied on self-reported sleep measures.

Furthermore, the main results show that, among other factors, perceived sleep quality was associated with mental health outcomes, consistent with previous research [14,46,47]. Sleep and mental health are closely intertwined, with sleep deprivation increasing the risk of developing mental health problems, which can then detrimentally impact academic performance [48]. Perceived sleep quality specifically influences measures of psychological distress and cannabis use, even when considering its interactions with drop-out intentions and a history of mental disorder diagnosis. Regarding drop-out intentions, results reveal that among students with medium/high intentions of leaving university, perceived sleep quality was significantly and substantially associated with perceived academic distress. This suggests that feeling well-rested might be particularly crucial for students already contemplating leaving university. The stress of a major decision, coupled with fatigue from poor sleep, may exacerbate existing academic pressures and significantly increase their distress levels. 

Perceived sleep quality, when considered alongside a lifetime diagnosis of a mental disorder, appeared to correlate with differences in cannabis consumption, as students dissatisfied with their sleep quality tended to report significantly higher scores. Given the increased vulnerability of students with a history of mental disorders to experiencing mental health problems in university [9], it is plausible that poor sleep quality could potentially contribute to some students’ decision to self-medicate in an attempt to improve sleep [49]. However, it is important to note that while low perceived sleep quality and resulting discomfort may suggest a link to cannabis self-medication to alleviate distress [50], further research is needed to confirm this hypothesis.

This study suggests a strong connection between sleep habits, substance use, and mental health in college students. Healthy sleep habits might be particularly important for students at risk of dropping out or using substances, as sleep problems and resulting distress may lead them to seek coping mechanisms like substance use [51]. Additionally, healthy sleep habits have the potential to be particularly beneficial for vulnerable students, such as those already diagnosed with a mental disorder, where substance use may further deteriorate their mental health [50].

While our study focused on examining the relationship between sleep and mental health outcomes among college freshmen, it is crucial to acknowledge the broader context of student well-being. Promoting good sleep could be an important component of a broader program aimed at improving student well-being. The university lifestyle can foster unhealthy behaviors like lack of exercise, which can further impact sleep quality and overall health [52]. Adequate sleep plays a crucial role in managing stress, which is a common challenge among college students due to academic, social, and extracurricular commitments [53]. Quality sleep can help reduce stress, improve coping skills, and enhance academic performance, potentially due to better memory, concentration, and problem-solving abilities [48,54,55].

While our study sheds light on the relationship between sleep and mental health outcomes among college freshmen, future research should continue to explore these relationships to develop more effective interventions and support mechanisms for promoting student well-being. Universities might consider strengthening their counseling services to provide adequate support for students struggling with stress and sleep issues. Promoting a culture of well-being can not only benefit students’ personal health and future prospects but also have significant positive implications from social and economic perspectives.

### Limitations

This study has some limitations, mainly concerning the generalizability of the results. Data were gathered only from freshmen at a single university in northern Italy, and voluntary participation may have introduced selection bias, as students who participated might differ from those who declined. Further studies including more representative samples and cross-cultural comparisons are therefore needed. The self-report nature of our assessments is a second limitation, potentially affecting the validity of the results. Additionally, while we used two measures to assess sleep (number of hours and perceived quality), employing a more comprehensive and standardized questionnaire would have allowed for a more accurate and detailed assessment, facilitating comparisons with other studies. Another limitation is that we focused on specific mental health outcomes, such as psychophysical well-being, university distress, substance use, and problematic internet use. These chosen outcomes, while providing a broad view of the emotional and psychological experiences of college students, may not capture the full spectrum of their mental health. Furthermore, the results regarding cannabis use should be interpreted with caution due to the low number of students using this substance and their reporting of medium/high-risk use. Social desirability bias might also have led to an underestimation of actual use.

## 5. Conclusions

This study contributes to understanding the role of sleep in the mental health of college freshmen by examining various mental health outcomes and interactions with sociodemographic and clinical factors. Our findings revealed a high prevalence of poor sleep quality and dissatisfaction among students. Notably, perceived sleep quality showed a strong association with psychological distress, particularly among those considering dropping out. Furthermore, among freshmen with the highest drop-out intentions, those reporting the most dissatisfaction with sleep quality also reported the highest cannabis use. These findings highlight the potential value of exploring interventions aimed at enhancing sleep quality and promoting healthy lifestyle choices among college students. Further research is warranted to gain a deeper understanding of the complex interaction that exists between sleep, mental health, and other sociodemographic, clinical, and academic factors.

## Figures and Tables

**Table 1:** Characteristics of the sample.

Variable	n	%
Relationship Status		
Single	405	56.2
With a partner	314	43.6
Missing	2	0.2
Employment status		
Student	473	65.6
Student worker	248	34.4
Field of Study		
Medicine and Pharmacy	325	45.1
Engineering and Agricultural	170	23.6
Economics	180	25.0
Law	46	6.4
Satisfaction with perceived quality of sleep		
Dissatisfied	258	35.8
Neither satisfied nor dissatisfied	170	23.6
Satisfied	293	40.6
Hours of sleep per night		
Less than 7 h	186	25.8
Equal or more than 7 h	532	73.8
Missing	3	0.4
ASSIST—Level of risky use of alcohol		
Low risk	638	88.5
Medium/high risk	83	11.5
ASSIST—Level of risky use of cannabis		
Low risk	670	92.9
Medium/high risk	51	7.1
Intentions to drop out of university		
Low intentions	514	71.3
Medium/high intentions	207	28.7
	Mean	SD
Hours of sleep per night	7.02	1.04
GHQ-12 total score	6.07	2.91
USS total score	14.43	8.39
IAUQ total score	12.43	9.43
ASSIST Alcohol Total Score	4.71	5.04
ASSIST Cannabis Total Score	0.84	3.33

**Table 2:** Multivariate analysis.

Effect	Pillai’s Trace	F	df_num_	df_den_	*p*	η_p_^2^
Gender	0.053	7.485	5.000	668.000	<0.001	0.053
Relationship status	0.002	0.223	5.000	668.000	0.953	0.002
Field of study	0.035	1.595	15.000	2010.000	0.067	0.012
Employment status	0.009	1.270	5.000	668.000	0.275	0.009
Sleep satisfaction	0.043	2.957	10.000	1338.000	0.001	0.022
Hours of sleep	0.003	0.377	5.000	668.000	0.865	0.003
Psychological support lifetime	0.017	2.280	5.000	668.000	0.045	0.017
Diagnosis of mental disorder lifetime	0.020	2.703	5.000	668.000	0.020	0.020
Drop-out intentions	0.084	12.225	5.000	668.000	<0.001	0.084
Gender × Sleep satisfaction	0.018	1.238	10.000	1338.000	0.262	0.009
Relationship status × Sleep satisfaction	0.016	1.048	10.000	1338.000	0.400	0.008
Field of study × Sleep satisfaction	0.053	1.193	30.000	3360.000	0.217	0.011
Employment status × Sleep satisfaction	0.026	1.781	10.000	1338.000	0.059	0.013
Psychological support lifetime × Sleep satisfaction	0.005	0.360	10.000	1338.000	0.963	0.003
Diagnosis of mental disorder lifetime × Sleep satisfaction	0.040	2.744	10.000	1338.000	0.002	0.020
Drop-out intentions × Sleep satisfaction	0.029	1.978	10.000	1338.000	0.032	0.015
Gender × Hours of sleep	0.014	1.953	5.000	668.000	0.084	0.014
Relationship status × Hours of sleep	0.009	1.187	5.000	668.000	0.314	0.009
Field of study × Hours of sleep	0.035	1.582	15.000	2010.000	0.071	0.012
Employment status × Hours of sleep	0.002	0.276	5.000	668.000	0.927	0.002
Psychological support lifetime × Hours of sleep	0.006	0.848	5.000	668.000	0.516	0.006
Diagnosis of mental disorder lifetime × Hours of sleep	0.001	0.168	5.000	668.000	0.974	0.001
Drop-out intentions × Hours of sleep	0.003	0.366	5.000	668.000	0.872	0.003

Multivariate analysis with Pillai’s Trace on collected variables, analyzing both main effects and interactions for sleep variables. η_p_^2^ = partial eta squared.

## Data Availability

The data that support the findings of this study are available upon request from the corresponding author.

## Supplementary Materials

The following supporting information can be downloaded at https://www.mdpi.com/article/10.3390/jcm13092626/s1, Table S1: Correlations between outcome variables; Table S2. Pairwise correlations satisfaction with sleep quality.
