# Supplementary material for: The Interplay of Sleep Quality, Mental Health, and Sociodemographic and Clinical Factors among Italian College Freshmen
Source: J Clin Med. 2024 Apr 29;13(9):2626. doi: 10.3390/jcm13092626 (PMC11084360; doi:10.3390/jcm13092626)
Supplement: Supplementary file 1 [file jcm-13-02626-s001.zip › Supplementary materials def.pdf]

Table S1. Correlations between outcome variables.

		GHQ-12 total score	USS total score	IAUQ total score	ASSIST alcohol total score	ASSIST cannabis total score
GHQ-12 total score	Pearson	1	.423**	.267**	.089*	.054
	Sig. (2-tailed)		<.001	<.001	.017	.147
	N	721	721	721	721	721
USS total score	Pearson	.423**	1	.427**	.187**	.128**
	Sig. (2-tailed)	<.001		<.001	<.001	<.001
	N	721	721	721	721	721
IAUQ total score	Pearson	.267**	.427**	1	.174**	.091*
	Sig. (2-tailed)	<.001	<.001		<.001	.014
	N	721	721	721	721	721
ASSIST_alcohol total score	Pearson	.089*	.187**	.174**	1	.305**
	Sig. (2-tailed)	.017	<.001	<.001		<.001
	N	721	721	721	721	721
ASSIST_cannabis total score	Pearson Correlation	.054	.128**	.091*	.305**	1
	Sig. (2-tailed)	.147	<.001	.014	<.001	
	N	721	721	721	721	721

**. Correlation is significant at the 0.01 level (2-tailed).

*. Correlation is significant at the 0.05 level (2-tailed).

Table S2. Pairwise correlations_satisfaction with sleep quality.

Pairwise Comparisons								
Dependent Variable	Independent Variable	Sleep satisfaction (I)	Sleep satisfaction (J)	Mean Difference (I-J)	Std. Error	Sig.[1]	95% Confidence Interval for Difference[1]	
							Lower Bound	Upper Bound
	Gender							
GHQ total score	male	dissatisfied	neutral	2.687	1.509	.226	-.935	6.310
			satisfied	2.626	1.543	.268	-1.077	6.328
		neutral	dissatisfied	-2.687	1.509	.226	-6.310	.935
			satisfied	-.062	1.518	1.000	-3.706	3.582
		satisfied	dissatisfied	-2.626	1.543	.268	-6.328	1.077
			neutral	.062	1.518	1.000	-3.582	3.706
	female	dissatisfied	neutral	3.897*	1.230	.005	.944	6.849
			satisfied	2.376	1.327	.221	-.809	5.560
		neutral	dissatisfied	-3.897*	1.230	.005	-6.849	-.944
			satisfied	-1.521	1.375	.808	-4.822	1.780
		satisfied	dissatisfied	-2.376	1.327	.221	-5.560	.809
			neutral	1.521	1.375	.808	-1.780	4.822
USS total score	male	dissatisfied	neutral	4.731	1.978	.051	-.017	9.478
			satisfied	4.303	2.022	.101	-.550	9.156
		neutral	dissatisfied	-4.731	1.978	.051	-9.478	.017
			satisfied	-.427	1.990	1.000	-5.204	4.349
		satisfied	dissatisfied	-4.303	2.022	.101	-9.156	.550
			neutral	.427	1.990	1.000	-4.349	5.204
	female	dissatisfied	neutral	2.781	1.613	.255	-1.089	6.651
			satisfied	4.095	1.739	.056	-.079	8.269
		neutral	dissatisfied	-2.781	1.613	.255	-6.651	1.089
			satisfied	1.314	1.803	1.000	-3.013	5.641
		satisfied	dissatisfied	-4.095	1.739	.056	-8.269	.079

			neutral	-1.314	1.803	1.000	-5.641	3.013
IAUQ total score	male	dissatisfied	neutral	-.434	2.477	1.000	-6.379	5.511
			satisfied	.991	2.532	1.000	-5.086	7.068
		neutral	dissatisfied	.434	2.477	1.000	-5.511	6.379
			satisfied	1.425	2.492	1.000	-4.556	7.406
		satisfied	dissatisfied	-.991	2.532	1.000	-7.068	5.086
			neutral	-1.425	2.492	1.000	-7.406	4.556
	female	dissatisfied	neutral	-.682	2.019	1.000	-5.528	4.164
			satisfied	1.665	2.178	1.000	-3.562	6.892
		neutral	dissatisfied	.682	2.019	1.000	-4.164	5.528
			satisfied	2.347	2.257	.897	-3.071	7.765
		satisfied	dissatisfied	-1.665	2.178	1.000	-6.892	3.562
			neutral	-2.347	2.257	.897	-7.765	3.071
ASSIST_alcool total score	male	dissatisfied	neutral	1.766	1.341	.565	-1.452	4.984
			satisfied	1.896	1.370	.501	-1.393	5.185
		neutral	dissatisfied	-1.766	1.341	.565	-4.984	1.452
			satisfied	.130	1.349	1.000	-3.108	3.367
		satisfied	dissatisfied	-1.896	1.370	.501	-5.185	1.393
			neutral	-.130	1.349	1.000	-3.367	3.108
	female	dissatisfied	neutral	1.486	1.093	.523	-1.137	4.109
			satisfied	1.010	1.179	1.000	-1.819	3.839
		neutral	dissatisfied	-1.486	1.093	.523	-4.109	1.137
			satisfied	-.476	1.222	1.000	-3.408	2.457
		satisfied	dissatisfied	-1.010	1.179	1.000	-3.839	1.819
			neutral	.476	1.222	1.000	-2.457	3.408
ASSIST_cannabis total score	male	dissatisfied	neutral	3.481*	.856	.000	1.425	5.536
			satisfied	3.046*	.875	.002	.946	5.147
		neutral	dissatisfied	-3.481*	.856	.000	-5.536	-1.425
			satisfied	-.434	.862	1.000	-2.502	1.633
		satisfied	dissatisfied	-3.046*	.875	.002	-5.147	-.946
			neutral	.434	.862	1.000	-1.633	2.502
	female	dissatisfied	neutral	2.117*	.698	.008	.441	3.792
			satisfied	1.345	.753	.223	-.462	3.152

		neutral	dissatisfied	-2.117*	.698	.008	-3.792	-.441
			satisfied	-.772	.780	.969	-2.645	1.101
		satisfied	dissatisfied	-1.345	.753	.223	-3.152	.462
			neutral	.772	.780	.969	-1.101	2.645
	Relationship status							
GHQ total score	single	dissatisfied	neutral	2.693	1.332	.131	-.504	5.890
			satisfied	1.595	1.417	.782	-1.805	4.995
		neutral	dissatisfied	-2.693	1.332	.131	-5.890	.504
			satisfied	-1.098	1.441	1.000	-4.557	2.361
		satisfied	dissatisfied	-1.595	1.417	.782	-4.995	1.805
			neutral	1.098	1.441	1.000	-2.361	4.557
	with a partner	dissatisfied	neutral	3.891*	1.377	.015	.587	7.195
			satisfied	3.406*	1.412	.048	.019	6.794
		neutral	dissatisfied	-3.891*	1.377	.015	-7.195	-.587
			satisfied	-.485	1.419	1.000	-3.891	2.921
		satisfied	dissatisfied	-3.406*	1.412	.048	-6.794	-.019
			neutral	.485	1.419	1.000	-2.921	3.891
USS total score	single	dissatisfied	neutral	2.850	1.746	.309	-1.340	7.041
			satisfied	3.709	1.857	.139	-.748	8.166
		neutral	dissatisfied	-2.850	1.746	.309	-7.041	1.340
			satisfied	.859	1.889	1.000	-3.676	5.393
		satisfied	dissatisfied	-3.709	1.857	.139	-8.166	.748
			neutral	-.859	1.889	1.000	-5.393	3.676
	with a partner	dissatisfied	neutral	4.661*	1.805	.030	.331	8.992
			satisfied	4.690*	1.850	.034	.249	9.130
		neutral	dissatisfied	-4.661*	1.805	.030	-8.992	-.331
			satisfied	.028	1.860	1.000	-4.436	4.492
		satisfied	dissatisfied	-4.690*	1.850	.034	-9.130	-.249
			neutral	-.028	1.860	1.000	-4.492	4.436
IAUQ total score	single	dissatisfied	neutral	-.333	2.186	1.000	-5.580	4.915
			satisfied	.371	2.325	1.000	-5.210	5.952
		neutral	dissatisfied	.333	2.186	1.000	-4.915	5.580
			satisfied	.704	2.366	1.000	-4.974	6.382

		satisfied	dissatisfied	-.371	2.325	1.000	-5.952	5.210
			neutral	-.704	2.366	1.000	-6.382	4.974
	with a partner	dissatisfied	neutral	-.784	2.260	1.000	-6.207	4.639
			satisfied	2.284	2.317	.974	-3.276	7.845
		neutral	dissatisfied	.784	2.260	1.000	-4.639	6.207
			satisfied	3.068	2.329	.565	-2.522	8.658
		satisfied	dissatisfied	-2.284	2.317	.974	-7.845	3.276
			neutral	-3.068	2.329	.565	-8.658	2.522
ASSIST_alcool total score	single	dissatisfied	neutral	1.212	1.183	.919	-1.629	4.052
			satisfied	1.569	1.259	.639	-1.452	4.590
		neutral	dissatisfied	-1.212	1.183	.919	-4.052	1.629
			satisfied	.357	1.281	1.000	-2.716	3.431
		satisfied	dissatisfied	-1.569	1.259	.639	-4.590	1.452
			neutral	-.357	1.281	1.000	-3.431	2.716
	with a partner	dissatisfied	neutral	2.040	1.223	.287	-.895	4.976
			satisfied	1.337	1.254	.860	-1.673	4.347
		neutral	dissatisfied	-2.040	1.223	.287	-4.976	.895
			satisfied	-.703	1.261	1.000	-3.729	2.323
		satisfied	dissatisfied	-1.337	1.254	.860	-4.347	1.673
			neutral	.703	1.261	1.000	-2.323	3.729
ASSIST_cannabis total score	single	dissatisfied	neutral	2.685*	.756	.001	.871	4.499
			satisfied	2.684*	.804	.003	.755	4.614
		neutral	dissatisfied	-2.685*	.756	.001	-4.499	-.871
			satisfied	-.001	.818	1.000	-1.964	1.962
		satisfied	dissatisfied	-2.684*	.804	.003	-4.614	-.755
			neutral	.001	.818	1.000	-1.962	1.964
	with a partner	dissatisfied	neutral	2.912*	.781	.001	1.037	4.787
			satisfied	1.707	.801	.100	-.215	3.629
		neutral	dissatisfied	-2.912*	.781	.001	-4.787	-1.037
			satisfied	-1.205	.805	.405	-3.138	.727
		satisfied	dissatisfied	-1.707	.801	.100	-3.629	.215
			neutral	1.205	.805	.405	-.727	3.138
	Field of study							

GHQ total score	Medicine & Pharmacy	dissatisfied	neutral	1.876	1.364	.509	-1.399	5.150
			satisfied	.406	1.478	1.000	-3.141	3.954
		neutral	dissatisfied	-1.876	1.364	.509	-5.150	1.399
			satisfied	-1.469	1.462	.945	-4.977	2.038
		satisfied	dissatisfied	-.406	1.478	1.000	-3.954	3.141
			neutral	1.469	1.462	.945	-2.038	4.977
	Engineering & Agricultural	dissatisfied	neutral	.785	1.386	1.000	-2.541	4.112
			satisfied	.860	1.635	1.000	-3.063	4.784
		neutral	dissatisfied	-.785	1.386	1.000	-4.112	2.541
			satisfied	.075	1.666	1.000	-3.924	4.074
		satisfied	dissatisfied	-.860	1.635	1.000	-4.784	3.063
			neutral	-.075	1.666	1.000	-4.074	3.924
	Economics	dissatisfied	neutral	4.687*	1.503	.006	1.080	8.295
			satisfied	2.969	1.553	.169	-.758	6.696
		neutral	dissatisfied	-4.687*	1.503	.006	-8.295	-1.080
			satisfied	-1.719	1.578	.829	-5.506	2.068
		satisfied	dissatisfied	-2.969	1.553	.169	-6.696	.758
			neutral	1.719	1.578	.829	-2.068	5.506
	Law	dissatisfied	neutral	5.820	2.487	.059	-.149	11.790
			satisfied	5.768	2.528	.069	-.300	11.835
		neutral	dissatisfied	-5.820	2.487	.059	-11.790	.149
			satisfied	-.053	2.421	1.000	-5.863	5.758
		satisfied	dissatisfied	-5.768	2.528	.069	-11.835	.300
			neutral	.053	2.421	1.000	-5.758	5.863
USS total score	Medicine & Pharmacy	dissatisfied	neutral	.919	1.788	1.000	-3.373	5.211
			satisfied	.614	1.937	1.000	-4.036	5.264
		neutral	dissatisfied	-.919	1.788	1.000	-5.211	3.373
			satisfied	-.305	1.916	1.000	-4.903	4.293
		satisfied	dissatisfied	-.614	1.937	1.000	-5.264	4.036
			neutral	.305	1.916	1.000	-4.293	4.903
	Engineering & Agricultural	dissatisfied	neutral	1.649	1.817	1.000	-2.711	6.010
			satisfied	3.818	2.143	.226	-1.324	8.961
		neutral	dissatisfied	-1.649	1.817	1.000	-6.010	2.711

			satisfied	2.169	2.184	.963	-3.073	7.411
		satisfied	dissatisfied	-3.818	2.143	.226	-8.961	1.324
			neutral	-2.169	2.184	.963	-7.411	3.073
	Economics	dissatisfied	neutral	5.480*	1.970	.017	.751	10.209
			satisfied	4.319	2.035	.103	-.566	9.204
		neutral	dissatisfied	-5.480*	1.970	.017	-10.209	-.751
			satisfied	-1.161	2.068	1.000	-6.125	3.804
		satisfied	dissatisfied	-4.319	2.035	.103	-9.204	.566
			neutral	1.161	2.068	1.000	-3.804	6.125
	Law	dissatisfied	neutral	6.975	3.260	.098	-.850	14.800
			satisfied	8.045*	3.314	.046	.091	15.999
		neutral	dissatisfied	-6.975	3.260	.098	-14.800	.850
			satisfied	1.070	3.173	1.000	-6.546	8.686
		satisfied	dissatisfied	-8.045*	3.314	.046	-15.999	-.091
			neutral	-1.070	3.173	1.000	-8.686	6.546
IAUQ total score	Medicine & Pharmacy	dissatisfied	neutral	-1.407	2.239	1.000	-6.782	3.967
			satisfied	-1.852	2.426	1.000	-7.675	3.970
		neutral	dissatisfied	1.407	2.239	1.000	-3.967	6.782
			satisfied	-.445	2.399	1.000	-6.202	5.312
		satisfied	dissatisfied	1.852	2.426	1.000	-3.970	7.675
			neutral	.445	2.399	1.000	-5.312	6.202
	Engineering & Agricultural	dissatisfied	neutral	-4.280	2.275	.181	-9.740	1.180
			satisfied	-.900	2.683	1.000	-7.339	5.540
		neutral	dissatisfied	4.280	2.275	.181	-1.180	9.740
			satisfied	3.380	2.735	.651	-3.184	9.944
		satisfied	dissatisfied	.900	2.683	1.000	-5.540	7.339
			neutral	-3.380	2.735	.651	-9.944	3.184
	Economics	dissatisfied	neutral	-.075	2.467	1.000	-5.996	5.847
			satisfied	1.790	2.549	1.000	-4.327	7.908
		neutral	dissatisfied	.075	2.467	1.000	-5.847	5.996
			satisfied	1.865	2.590	1.000	-4.351	8.081
		satisfied	dissatisfied	-1.790	2.549	1.000	-7.908	4.327
			neutral	-1.865	2.590	1.000	-8.081	4.351

	Law	dissatisfied	neutral	3.529	4.083	1.000	-6.269	13.328
			satisfied	6.273	4.150	.393	-3.687	16.233
		neutral	dissatisfied	-3.529	4.083	1.000	-13.328	6.269
			satisfied	2.744	3.974	1.000	-6.793	12.280
		satisfied	dissatisfied	-6.273	4.150	.393	-16.233	3.687
			neutral	-2.744	3.974	1.000	-12.280	6.793
ASSIST_alcool total score	Medicine & Pharmacy	dissatisfied	neutral	2.142	1.212	.233	-.767	5.052
			satisfied	1.892	1.313	.450	-1.260	5.043
		neutral	dissatisfied	-2.142	1.212	.233	-5.052	.767
			satisfied	-.251	1.298	1.000	-3.367	2.866
		satisfied	dissatisfied	-1.892	1.313	.450	-5.043	1.260
			neutral	.251	1.298	1.000	-2.866	3.367
	Engineering & Agricultural	dissatisfied	neutral	1.254	1.231	.926	-1.701	4.210
			satisfied	.047	1.452	1.000	-3.439	3.532
		neutral	dissatisfied	-1.254	1.231	.926	-4.210	1.701
			satisfied	-1.207	1.480	1.000	-4.760	2.345
		satisfied	dissatisfied	-.047	1.452	1.000	-3.532	3.439
			neutral	1.207	1.480	1.000	-2.345	4.760
	Economics	dissatisfied	neutral	1.582	1.335	.710	-1.623	4.787
			satisfied	1.574	1.380	.763	-1.737	4.885
		neutral	dissatisfied	-1.582	1.335	.710	-4.787	1.623
			satisfied	-.008	1.402	1.000	-3.373	3.356
		satisfied	dissatisfied	-1.574	1.380	.763	-4.885	1.737
			neutral	.008	1.402	1.000	-3.356	3.373
	Law	dissatisfied	neutral	1.525	2.210	1.000	-3.778	6.829
			satisfied	2.300	2.246	.919	-3.091	7.691
		neutral	dissatisfied	-1.525	2.210	1.000	-6.829	3.778
			satisfied	.775	2.151	1.000	-4.387	5.937
		satisfied	dissatisfied	-2.300	2.246	.919	-7.691	3.091
			neutral	-.775	2.151	1.000	-5.937	4.387
ASSIST_cannabis total score	Medicine & Pharmacy	dissatisfied	neutral	3.280*	.774	.000	1.422	5.138
			satisfied	3.367*	.839	.000	1.354	5.380
		neutral	dissatisfied	-3.280*	.774	.000	-5.138	-1.422

			satisfied	.086	.829	1.000	-1.904	2.077
		satisfied	dissatisfied	-3.367*	.839	.000	-5.380	-1.354
			neutral	-.086	.829	1.000	-2.077	1.904
	Engineering & Agricultural	dissatisfied	neutral	2.153*	.786	.019	.266	4.041
			satisfied	.793	.928	1.000	-1.433	3.019
		neutral	dissatisfied	-2.153*	.786	.019	-4.041	-.266
			satisfied	-1.361	.945	.452	-3.630	.909
		satisfied	dissatisfied	-.793	.928	1.000	-3.019	1.433
			neutral	1.361	.945	.452	-.909	3.630
	Economics	dissatisfied	neutral	2.750*	.853	.004	.703	4.797
			satisfied	2.736*	.881	.006	.622	4.851
		neutral	dissatisfied	-2.750*	.853	.004	-4.797	-.703
			satisfied	-.014	.895	1.000	-2.163	2.135
		satisfied	dissatisfied	-2.736*	.881	.006	-4.851	-.622
			neutral	.014	.895	1.000	-2.135	2.163
	Law	dissatisfied	neutral	3.011	1.411	.100	-.377	6.398
			satisfied	1.887	1.435	.567	-1.556	5.330
		neutral	dissatisfied	-3.011	1.411	.100	-6.398	.377
			satisfied	-1.124	1.374	1.000	-4.421	2.173
		satisfied	dissatisfied	-1.887	1.435	.567	-5.330	1.556
			neutral	1.124	1.374	1.000	-2.173	4.421
	Employment status							
GHQ total score	Student worker	dissatisfied	neutral	3.224	1.382	.060	-.093	6.541
			satisfied	1.839	1.463	.628	-1.673	5.350
		neutral	dissatisfied	-3.224	1.382	.060	-6.541	.093
			satisfied	-1.385	1.501	1.000	-4.987	2.216
		satisfied	dissatisfied	-1.839	1.463	.628	-5.350	1.673
			neutral	1.385	1.501	1.000	-2.216	4.987
	Student	dissatisfied	neutral	3.360*	1.340	.037	.144	6.577
			satisfied	3.163	1.399	.072	-.195	6.520
		neutral	dissatisfied	-3.360*	1.340	.037	-6.577	-.144
			satisfied	-.198	1.408	1.000	-3.578	3.182
		satisfied	dissatisfied	-3.163	1.399	.072	-6.520	.195

			neutral	.198	1.408	1.000	-3.182	3.578
USS total score	Student worker	dissatisfied	neutral	1.799	1.812	.963	-2.549	6.147
			satisfied	1.804	1.918	1.000	-2.799	6.406
		neutral	dissatisfied	-1.799	1.812	.963	-6.147	2.549
			satisfied	.005	1.967	1.000	-4.716	4.725
		satisfied	dissatisfied	-1.804	1.918	1.000	-6.406	2.799
			neutral	-.005	1.967	1.000	-4.725	4.716
	Student	dissatisfied	neutral	5.712*	1.757	.004	1.496	9.928
			satisfied	6.594*	1.834	.001	2.194	10.995
		neutral	dissatisfied	-5.712*	1.757	.004	-9.928	-1.496
			satisfied	.882	1.846	1.000	-3.548	5.313
		satisfied	dissatisfied	-6.594*	1.834	.001	-10.995	-2.194
			neutral	-.882	1.846	1.000	-5.313	3.548
IAUQ total score	Student worker	dissatisfied	neutral	-2.388	2.269	.879	-7.833	3.056
			satisfied	-.725	2.401	1.000	-6.488	5.038
		neutral	dissatisfied	2.388	2.269	.879	-3.056	7.833
			satisfied	1.663	2.463	1.000	-4.248	7.574
		satisfied	dissatisfied	.725	2.401	1.000	-5.038	6.488
			neutral	-1.663	2.463	1.000	-7.574	4.248
	Student	dissatisfied	neutral	1.272	2.200	1.000	-4.007	6.551
			satisfied	3.381	2.296	.424	-2.130	8.891
		neutral	dissatisfied	-1.272	2.200	1.000	-6.551	4.007
			satisfied	2.109	2.312	1.000	-3.439	7.657
		satisfied	dissatisfied	-3.381	2.296	.424	-8.891	2.130
			neutral	-2.109	2.312	1.000	-7.657	3.439
ASSIST_alcool total score	Student worker	dissatisfied	neutral	.903	1.228	1.000	-2.043	3.850
			satisfied	1.524	1.300	.725	-1.596	4.643
		neutral	dissatisfied	-.903	1.228	1.000	-3.850	2.043
			satisfied	.620	1.333	1.000	-2.579	3.820
		satisfied	dissatisfied	-1.524	1.300	.725	-4.643	1.596
			neutral	-.620	1.333	1.000	-3.820	2.579
	Student	dissatisfied	neutral	2.349	1.191	.147	-.509	5.206
			satisfied	1.383	1.243	.799	-1.600	4.365

		neutral	dissatisfied	-2.349	1.191	.147	-5.206	.509
			satisfied	-.966	1.251	1.000	-3.969	2.037
		satisfied	dissatisfied	-1.383	1.243	.799	-4.365	1.600
			neutral	.966	1.251	1.000	-2.037	3.969
ASSIST_cannabis total score	Student worker	dissatisfied	neutral	2.969*	.784	.001	1.086	4.851
			satisfied	2.557*	.830	.006	.565	4.550
		neutral	dissatisfied	-2.969*	.784	.001	-4.851	-1.086
			satisfied	-.411	.851	1.000	-2.455	1.632
		satisfied	dissatisfied	-2.557*	.830	.006	-4.550	-.565
			neutral	.411	.851	1.000	-1.632	2.455
	Student	dissatisfied	neutral	2.629*	.760	.002	.804	4.454
			satisfied	1.834	.794	.063	-.071	3.739
		neutral	dissatisfied	-2.629*	.760	.002	-4.454	-.804
			satisfied	-.795	.799	.961	-2.712	1.123
		satisfied	dissatisfied	-1.834	.794	.063	-3.739	.071
			neutral	.795	.799	.961	-1.123	2.712
	Lifetime psychological support							
GHQ total score	yes	dissatisfied	neutral	3.254	1.374	.055	-.045	6.552
			satisfied	1.810	1.403	.592	-1.557	5.177
		neutral	dissatisfied	-3.254	1.374	.055	-6.552	.045
			satisfied	-1.444	1.389	.897	-4.776	1.889
		satisfied	dissatisfied	-1.810	1.403	.592	-5.177	1.557
			neutral	1.444	1.389	.897	-1.889	4.776
	no	dissatisfied	neutral	3.330	1.424	.059	-.086	6.747
			satisfied	3.191	1.497	.100	-.401	6.784
		neutral	dissatisfied	-3.330	1.424	.059	-6.747	.086
			satisfied	-.139	1.552	1.000	-3.865	3.586
		satisfied	dissatisfied	-3.191	1.497	.100	-6.784	.401
			neutral	.139	1.552	1.000	-3.586	3.865
USS total score	yes	dissatisfied	neutral	3.919	1.801	.090	-.404	8.243
			satisfied	3.912	1.839	.101	-.501	8.326

		neutral	dissatisfied	-3.919	1.801	.090	-8.243	.404
			satisfied	-.007	1.820	1.000	-4.375	4.362
		satisfied	dissatisfied	-3.912	1.839	.101	-8.326	.501
			neutral	.007	1.820	1.000	-4.362	4.375
	no	dissatisfied	neutral	3.593	1.866	.164	-.886	8.071
			satisfied	4.486	1.962	.068	-.223	9.195
		neutral	dissatisfied	-3.593	1.866	.164	-8.071	.886
			satisfied	.894	2.035	1.000	-3.990	5.777
		satisfied	dissatisfied	-4.486	1.962	.068	-9.195	.223
			neutral	-.894	2.035	1.000	-5.777	3.990
IAUQ total score	yes	dissatisfied	neutral	-.621	2.256	1.000	-6.034	4.793
			satisfied	1.234	2.303	1.000	-4.292	6.761
		neutral	dissatisfied	.621	2.256	1.000	-4.793	6.034
			satisfied	1.855	2.279	1.000	-3.615	7.325
		satisfied	dissatisfied	-1.234	2.303	1.000	-6.761	4.292
			neutral	-1.855	2.279	1.000	-7.325	3.615
	no	dissatisfied	neutral	-.496	2.337	1.000	-6.103	5.112
			satisfied	1.421	2.457	1.000	-4.475	7.318
		neutral	dissatisfied	.496	2.337	1.000	-5.112	6.103
			satisfied	1.917	2.548	1.000	-4.198	8.032
		satisfied	dissatisfied	-1.421	2.457	1.000	-7.318	4.475
			neutral	-1.917	2.548	1.000	-8.032	4.198
ASSIST_alcool total score	yes	dissatisfied	neutral	1.567	1.221	.599	-1.363	4.498
			satisfied	1.011	1.246	1.000	-1.980	4.003
		neutral	dissatisfied	-1.567	1.221	.599	-4.498	1.363
			satisfied	-.556	1.234	1.000	-3.517	2.405
		satisfied	dissatisfied	-1.011	1.246	1.000	-4.003	1.980
			neutral	.556	1.234	1.000	-2.405	3.517
	no	dissatisfied	neutral	1.685	1.265	.550	-1.351	4.720
			satisfied	1.895	1.330	.464	-1.297	5.086
		neutral	dissatisfied	-1.685	1.265	.550	-4.720	1.351
			satisfied	.210	1.379	1.000	-3.100	3.520
		satisfied	dissatisfied	-1.895	1.330	.464	-5.086	1.297

			neutral	-.210	1.379	1.000	-3.520	3.100
ASSIST_cannabis total score	yes	dissatisfied	neutral	2.524*	.780	.004	.652	4.395
			satisfied	1.647	.796	.117	-.263	3.558
		neutral	dissatisfied	-2.524*	.780	.004	-4.395	-.652
			satisfied	-.877	.788	.799	-2.768	1.014
		satisfied	dissatisfied	-1.647	.796	.117	-3.558	.263
			neutral	.877	.788	.799	-1.014	2.768
	no	dissatisfied	neutral	3.073*	.808	.000	1.135	5.012
			satisfied	2.744*	.849	.004	.706	4.783
		neutral	dissatisfied	-3.073*	.808	.000	-5.012	-1.135
			satisfied	-.329	.881	1.000	-2.443	1.785
		satisfied	dissatisfied	-2.744*	.849	.004	-4.783	-.706
			neutral	.329	.881	1.000	-1.785	2.443
	Lifetime diagnosis of mental disorder							
GHQ total score	yes	dissatisfied	neutral	4.204	2.276	.195	-1.257	9.666
			satisfied	4.178	2.387	.242	-1.551	9.908
		neutral	dissatisfied	-4.204	2.276	.195	-9.666	1.257
			satisfied	-.026	2.445	1.000	-5.893	5.841
		satisfied	dissatisfied	-4.178	2.387	.242	-9.908	1.551
			neutral	.026	2.445	1.000	-5.841	5.893
	no	dissatisfied	neutral	2.380*	.868	.019	.297	4.463
			satisfied	.823	.885	1.000	-1.301	2.947
		neutral	dissatisfied	-2.380*	.868	.019	-4.463	-.297
			satisfied	-1.557	.856	.209	-3.612	.499
		satisfied	dissatisfied	-.823	.885	1.000	-2.947	1.301
			neutral	1.557	.856	.209	-.499	3.612
USS total score	yes	dissatisfied	neutral	2.710	2.983	1.000	-4.449	9.869
			satisfied	6.153	3.129	.149	-1.357	13.664
		neutral	dissatisfied	-2.710	2.983	1.000	-9.869	4.449
			satisfied	3.443	3.204	.849	-4.247	11.133
		satisfied	dissatisfied	-6.153	3.129	.149	-13.664	1.357
			neutral	-3.443	3.204	.849	-11.133	4.247

	no	dissatisfied	neutral	4.801*	1.138	.000	2.071	7.531
			satisfied	2.245	1.160	.160	-.539	5.030
		neutral	dissatisfied	-4.801*	1.138	.000	-7.531	-2.071
			satisfied	-2.556	1.123	.069	-5.250	.138
		satisfied	dissatisfied	-2.245	1.160	.160	-5.030	.539
			neutral	2.556	1.123	.069	-.138	5.250
IAUQ total score	yes	dissatisfied	neutral	-4.480	3.735	.692	-13.444	4.484
			satisfied	1.342	3.918	1.000	-8.062	10.746
		neutral	dissatisfied	4.480	3.735	.692	-4.484	13.444
			satisfied	5.822	4.012	.442	-3.808	15.451
		satisfied	dissatisfied	-1.342	3.918	1.000	-10.746	8.062
			neutral	-5.822	4.012	.442	-15.451	3.808
	no	dissatisfied	neutral	3.364	1.424	.055	-.055	6.782
			satisfied	1.314	1.453	1.000	-2.173	4.801
		neutral	dissatisfied	-3.364	1.424	.055	-6.782	.055
			satisfied	-2.050	1.406	.436	-5.423	1.324
		satisfied	dissatisfied	-1.314	1.453	1.000	-4.801	2.173
			neutral	2.050	1.406	.436	-1.324	5.423
ASSIST_alcool total score	yes	dissatisfied	neutral	3.884	2.022	.165	-.968	8.736
			satisfied	2.474	2.121	.732	-2.617	7.564
		neutral	dissatisfied	-3.884	2.022	.165	-8.736	.968
			satisfied	-1.411	2.172	1.000	-6.623	3.801
		satisfied	dissatisfied	-2.474	2.121	.732	-7.564	2.617
			neutral	1.411	2.172	1.000	-3.801	6.623
	no	dissatisfied	neutral	-.632	.771	1.000	-2.483	1.218
			satisfied	.433	.786	1.000	-1.455	2.320
		neutral	dissatisfied	.632	.771	1.000	-1.218	2.483
			satisfied	1.065	.761	.486	-.761	2.891
		satisfied	dissatisfied	-.433	.786	1.000	-2.320	1.455
			neutral	-1.065	.761	.486	-2.891	.761
ASSIST_cannabis total score	yes	dissatisfied	neutral	5.109*	1.291	.000	2.011	8.208
			satisfied	4.477*	1.355	.003	1.226	7.728
		neutral	dissatisfied	-5.109*	1.291	.000	-8.208	-2.011

			satisfied	-.632	1.387	1.000	-3.961	2.697
		satisfied	dissatisfied	-4.477*	1.355	.003	-7.728	-1.226
			neutral	.632	1.387	1.000	-2.697	3.961
	no	dissatisfied	neutral	.488	.492	.967	-.694	1.670
			satisfied	-.086	.502	1.000	-1.291	1.120
		neutral	dissatisfied	-.488	.492	.967	-1.670	.694
			satisfied	-.573	.486	.715	-1.740	.593
		satisfied	dissatisfied	.086	.502	1.000	-1.120	1.291
			neutral	.573	.486	.715	-.593	1.740
	Intention to dropout from university							
GHQ total score	low intentions	dissatisfied	neutral	3.308*	1.265	.027	.272	6.344
			satisfied	2.164	1.337	.318	-1.044	5.372
		neutral	dissatisfied	-3.308*	1.265	.027	-6.344	-.272
			satisfied	-1.144	1.339	1.000	-4.358	2.070
		satisfied	dissatisfied	-2.164	1.337	.318	-5.372	1.044
			neutral	1.144	1.339	1.000	-2.070	4.358
	medium/high intentions	dissatisfied	neutral	3.276	1.487	.084	-.292	6.844
			satisfied	2.837	1.511	.183	-.790	6.464
		neutral	dissatisfied	-3.276	1.487	.084	-6.844	.292
			satisfied	-.439	1.575	1.000	-4.219	3.341
		satisfied	dissatisfied	-2.837	1.511	.183	-6.464	.790
			neutral	.439	1.575	1.000	-3.341	4.219
USS total score	low intentions	dissatisfied	neutral	2.411	1.658	.439	-1.569	6.390
			satisfied	.887	1.752	1.000	-3.318	5.092
		neutral	dissatisfied	-2.411	1.658	.439	-6.390	1.569
			satisfied	-1.524	1.755	1.000	-5.737	2.689
		satisfied	dissatisfied	-.887	1.752	1.000	-5.092	3.318
			neutral	1.524	1.755	1.000	-2.689	5.737
	medium/high intentions	dissatisfied	neutral	5.101*	1.949	.027	.424	9.778
			satisfied	7.512*	1.981	.000	2.758	12.265
		neutral	dissatisfied	-5.101*	1.949	.027	-9.778	-.424
			satisfied	2.411	2.065	.730	-2.544	7.366

		satisfied	dissatisfied	-7.512*	1.981	.000	-12.265	-2.758
			neutral	-2.411	2.065	.730	-7.366	2.544
IAUQ total score	low intentions	dissatisfied	neutral	-1.223	2.076	1.000	-6.205	3.760
			satisfied	.620	2.194	1.000	-4.646	5.885
		neutral	dissatisfied	1.223	2.076	1.000	-3.760	6.205
			satisfied	1.842	2.198	1.000	-3.433	7.117
		satisfied	dissatisfied	-.620	2.194	1.000	-5.885	4.646
			neutral	-1.842	2.198	1.000	-7.117	3.433
	medium/high intentions	dissatisfied	neutral	.106	2.440	1.000	-5.750	5.963
			satisfied	2.036	2.480	1.000	-3.917	7.989
		neutral	dissatisfied	-.106	2.440	1.000	-5.963	5.750
			satisfied	1.930	2.585	1.000	-4.275	8.134
		satisfied	dissatisfied	-2.036	2.480	1.000	-7.989	3.917
			neutral	-1.930	2.585	1.000	-8.134	4.275
ASSIST_alcool total score	low intentions	dissatisfied	neutral	1.809	1.124	.324	-.888	4.506
			satisfied	1.446	1.188	.671	-1.404	4.296
		neutral	dissatisfied	-1.809	1.124	.324	-4.506	.888
			satisfied	-.363	1.190	1.000	-3.218	2.492
		satisfied	dissatisfied	-1.446	1.188	.671	-4.296	1.404
			neutral	.363	1.190	1.000	-2.492	3.218
	medium/high intentions	dissatisfied	neutral	1.443	1.321	.825	-1.727	4.613
			satisfied	1.460	1.343	.832	-1.762	4.682
		neutral	dissatisfied	-1.443	1.321	.825	-4.613	1.727
			satisfied	.017	1.399	1.000	-3.341	3.375
		satisfied	dissatisfied	-1.460	1.343	.832	-4.682	1.762
			neutral	-.017	1.399	1.000	-3.375	3.341
ASSIST_cannabis total score	low intentions	dissatisfied	neutral	2.458*	.718	.002	.736	4.181
			satisfied	1.921*	.758	.035	.101	3.742
		neutral	dissatisfied	-2.458*	.718	.002	-4.181	-.736
			satisfied	-.537	.760	1.000	-2.360	1.287
		satisfied	dissatisfied	-1.921*	.758	.035	-3.742	-.101
			neutral	.537	.760	1.000	-1.287	2.360
	medium/high	dissatisfied	neutral	3.139*	.844	.001	1.114	5.164

intentions		satisfied	2.470*	.857	.012	.412	4.528
	neutral	dissatisfied	-3.139*	.844	.001	-5.164	-1.114
		satisfied	-.669	.894	1.000	-2.814	1.476
	satisfied	dissatisfied	-2.470*	.857	.012	-4.528	-.412
		neutral	.669	.894	1.000	-1.476	2.814

Based on estimated marginal means

*. The mean difference is significant at the .05 level.

[1]. Adjustment for multiple comparisons: Bonferroni.
